# The Role of Single-Cell Technology in the Study and Control of Infectious Diseases

**DOI:** 10.3390/cells9061440

**Published:** 2020-06-10

**Authors:** Weikang Nicholas Lin, Matthew Zirui Tay, Ri Lu, Yi Liu, Chia-Hung Chen, Lih Feng Cheow

**Affiliations:** 1 Department of Biomedical Engineering, National University of Singapore, Singapore 119007, Singapore; e0223119@u.nus.edu (W.N.L.); yi.liu@nus.edu.sg (Y.L.); 2 Singapore Immunology Network (SIgN), Agency for Science, Technology and Research (A∗STAR), Singapore 138648, Singapore; matthew_tay@immunol.a-star.edu.sg; 3 NUS Graduate School for Integrated Sciences and Engineering, Singapore 119007, Singapore; luri@u.nus.edu; 4 Department of Biomedical Engineering, City University of Hong Kong, 83 Tat Chee Avenue, Kowloon Tong 999077, Hong Kong SAR, China; chiachen@cityu.edu.hk; 5 Institute for Health Innovation & Technology (iHealthtech), Singapore 117599, Singapore

**Keywords:** single cell, infectious disease, pathophysiology, therapeutics, diagnostics

## Abstract

The advent of single-cell research in the recent decade has allowed biological studies at an unprecedented resolution and scale. In particular, single-cell analysis techniques such as Next-Generation Sequencing (NGS) and Fluorescence-Activated Cell Sorting (FACS) have helped show substantial links between cellular heterogeneity and infectious disease progression. The extensive characterization of genomic and phenotypic biomarkers, in addition to host–pathogen interactions at the single-cell level, has resulted in the discovery of previously unknown infection mechanisms as well as potential treatment options. In this article, we review the various single-cell technologies and their applications in the ongoing fight against infectious diseases, as well as discuss the potential opportunities for future development.

## 1. Introduction

Five months since the first reported infection cluster, COVID-19 has turned into a vicious worldwide pandemic that infected more than 3.6 million people and caused over 250,000 deaths [1]. The pandemic will also have large spillover effects in terms of economic damage both in the form of healthcare costs and in monetary losses from the disruption of global supply chains, with world trade expected to fall between 13% and 32% in 2020 [2]. The COVID-19 pandemic serves as a grim reminder that infectious disease is, and will always be, a major threat to the continued existence of mankind.

To date, there are about 1400 microorganisms known to be pathogenic to humans. These pathogens can be broadly classified as viral, bacterial, fungal, and parasitic pathogens [3]. In particular, there have been 3 infectious diseases that have been persistently difficult to eradicate, namely, Human Immunodeficiency Virus and Acquired Immune Deficiency Syndrome (HIV/AIDS), tuberculosis, and malaria. AIDS, due to HIV, is responsible for nearly 1 million deaths per year [4]. The death toll from tuberculosis, caused by Mycobacterium tuberculosis (MTB) bacteria, is the highest amongst all infectious diseases, which is a problem that is exacerbated by the rise of antimicrobial resistance variants of the disease [4]. Malaria, a parasitic infection, has afflicted humans for thousands of years and continues to do so today [5]. In light of the above-mentioned examples, among other infectious diseases, further efforts have to be directed for the continued management of the global burden of these diseases.

The COVID-19 pandemic has highlighted many questions that are relevant in the context of infectious disease as a whole. Why are certain people more susceptible to infections? Why are some infected individuals asymptomatic or display only mild symptoms? Why are there differences in terms of disease progression and outcomes among patients? This diverse response to infection could be explained by the interactions of inherently heterogeneous populations of pathogens, host cells, and immune cells. However, discerning this heterogeneity is difficult in conventional bulk analyses, as they fail to recognize the following: (1) the genomic variability of pathogens, (2) the coexistence and interactions of infected host cells and bystanders, and (3) the diverse functional roles of immune surveillance participants. Aside from the limited resolving power in pathophysiological studies, bulk analyses often fall short in terms of the level of precision and the amount of derived information needed for early diagnostics and high-efficacy vaccine development against infectious diseases.

Just as how microscopy revolutionized our understanding of biology, the enhanced resolution, precision, and breadth of information offered by single-cell technologies has brought an exciting overhaul to our perception of infectious diseases in recent years. The use of single-cell genomics, transcriptomics, proteomics, and epigenetics (referred to as omics altogether in this article) has flourished in many areas of the battlefield against infectious diseases. Table 1 presents commonly used single-cell technologies in infectious disease studies alongside several of other non-single-cell systems. A scoring heatmap is used to represent the complexity of information in various aspects that they can provide (e.g., genetic, epigenetic, proteomic, spatial). The heatmap also provides an overall ranking of throughput, cost, and downstream assay compatibility amongst all the listed techniques. Hence, it serves as a general guide for future users to select the methods that match their desired outputs. For instance, if the primary target for the study is to collect genetic information (e.g., analysis of invading virus gene heterogeneity in single host cell), single-cell sequencing might be the best candidate. Likewise, if the focus is on proteomics orchestrating the immune responses, mass cytometry can furnish the most detailed insight. For infectious disease models that involve the interplay of genomics and proteomics, both CITE-seq and REAP-seq can be the suitable candidates. While flow cytometry has the highest throughput and lowest cost per experiment amongst the listed single-cell methods, it yields very limited aspects of information. Other single-cell assays capable of providing more complex data typically come at the expense of a decreased throughput and increased cost. It is also worth mentioning that microfluidics has made great success in boosting the throughput and cost efficiency of existing single-cell assays. One prominent example would be the transition of well plate systems into microchambers or microdroplets, which ultimately reduces the required amount of reagent required per experiment and in turn reduces costs.

In this review, we identified infection pathophysiology, therapeutic discovery, and disease diagnostics as three major areas in which single-cell omics has contributed substantially in the past decade. In pathophysiological studies of infectious disease, single-cell omics offer excellent spatial–temporal resolution that help to not only reconstruct the uneven subcellular distribution of pathogen across the entire host cell population, but also reveal the sequence of immune events accompanied by the change of immune cell profiles. Single-cell omics also extrapolates meaningful molecular details that describe the dynamic host–pathogen interplay and immune activation. Furthermore, single-cell omics identifies the rare molecules and cell subtypes that exhibit significant functionality in the pathogen–host immune interactions. Insights in fundamental pathophysiology naturally have spillover benefits for translational science, such as in vaccine development where single-cell omics has the capability to enhance the discovery of mechanistic correlates of protection through multi-parameter measurements of the immune state with respect to disease, and it enables precision quality control checkpoints to aid the evaluation of vaccine efficacy. In the field of antibody discovery, single-cell omics can simultaneously interrogate antigen specificity and recover the B cell receptor gene sequences, which in turn shortens the previously prolonged and labor-intensive research cycle in the search for effective therapeutic or diagnostic antibodies. In the application of infectious disease diagnostics, single-cell omics are on the verge of practical clinical deployment, as demonstrated by some examples of automated and miniaturized devices. The diagnostic power of single-cell omics can be further enhanced by incorporating digital assays or integrating with other label free single-cell technologies. While there are many merits of single-cell analysis, we also discuss the new sets of challenges that need to be addressed in these systems. Finally, we will conclude with our insight on future prospects of single-cell research in infectious disease and highlight several emerging single-cell technologies that may further enrich our arsenal against infections.

## 2. Uncovering Infection Pathophysiology

Understanding the pathophysiology of infection is critical to the rational design of prophylactic and therapeutic strategies to tackle infectious diseases. The course of infection, determined by the encounter of pathogens and host cells, is often measured as population-averaged results, leaving the important cell-to-cell heterogeneity out of the picture. The heterogeneity arises from both the pathogens and the infected cells. For example, pathogen heterogeneity can be reflected in the case of viruses, as a mixture of mutated viral particles displaying different infection ability [6], or in the cases of bacteria, as a population of cells having different resistance to the same antibiotics [7]. Host cellular heterogeneity is a combined result of variances in metabolism, composition, activation status, cell cycle, or infection history [6]. Recent advances in single-cell analysis provide an attractive approach to probe the cellular population diversity and characterize infection pathophysiology at single-cell resolution. In this section, we will review how the recent advancement of single-cell technologies has helped deepen the understanding of pathogen and host cell heterogeneity and how the complex immune system reacts against infectious pathogens, with a focus on the contributions of single-cell sequencing.

### 2.1. Pathogen Heterogeneity

Pathogen heterogeneity can be inherent or as a result of heterogeneous host–pathogen interactions. It is a favorable feature for pathogens because varied genomic sequences or functional properties enable immune evasion, colonization in novel hosts, and drug resistance acquisition; therefore, they increase the possibility of survival. Besides, stochastic fluctuation in biochemical reactions may also contribute to cell-to-cell variability. Single-cell technologies provide high-resolution insights into different aspects of intracellular pathogen replication.

One area of virology that has benefited from the enhanced resolution of single-cell technologies is the study of variation in infection across single cells and the reasons for such variation. In the study by Heldt et al., cells were infected in a population, isolated into microwells, and incubated. The supernatant was subjected to viral plaques measurement, and viral RNA was quantified from lysed single infected cells [8]. It was shown that cells infected by influenza A virus (IAV) under the same conditions produced largely heterogenous progeny virus titers, ranging from 1 to 970 plaque-forming units (PFU) and intracellular viral RNA (vRNA) levels varied three orders of magnitude. Similarly, using scRNA-seq, another study determined the percentage of viral transcripts in the total mRNA generated from IAV-infected cells, and it revealed that while most cells contained less than 1% of viral transcripts, some cells generated more than 50%, demonstrating infection heterogeneity from the angle of viral load [9]. Reasons for this variation can be further explored through the use of high-throughput imaging technology. For instance, Akpninar et al. used virus expressing red fluorescence protein (RFP) to study the effect of defective interfering particles (DIP) on viral infection kinetics. DIP are noninfectious progeny particles lacking genes essential for replication, and they are commonly produced during infection due to the high mutation rate. When participating in infection along with viable viral particles, they compete for host cellular machinery and result in viral replication inhibition. In this study, cells in a bulk population were infected with a mixture of vesicular stomatitis virus (VSV) expressing RFP and VSV-DIPs, and they were either untreated or isolated by serial dilution. RFP expression was observed during incubation as a surrogate for viral replication levels. The results showed that DIP inhibited viral replication 10 times more on single cells, suggesting that the inhibition of viral replication is mitigated by cell–cell interactions when infection happens in a population [10].

**Figure 1:**
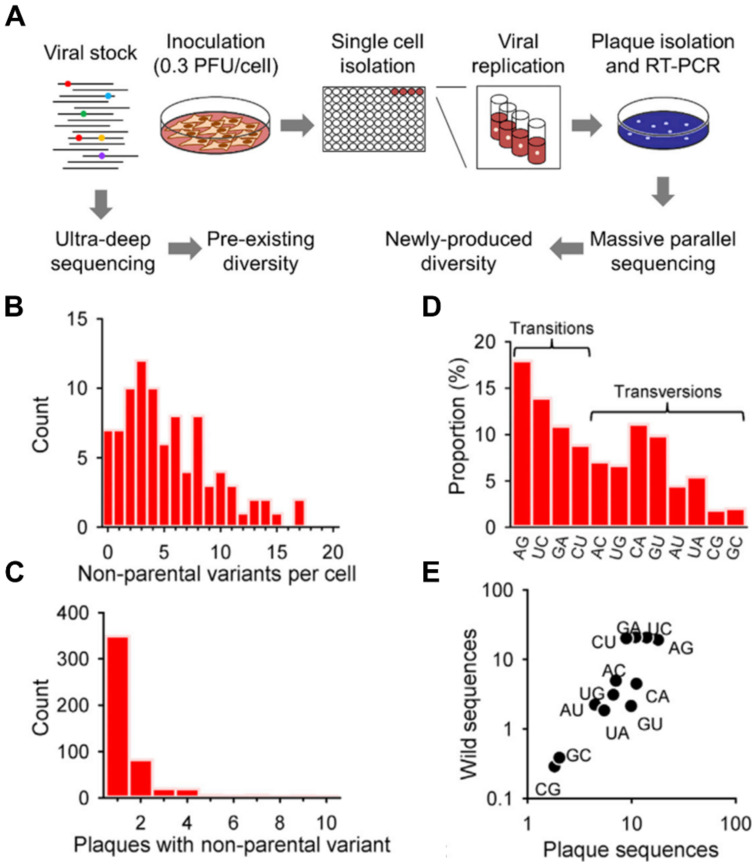
Pathogen heterogeneity revealed by single-cell analysis. (**A**) Schematic of experimental setup for sequencing single-cell bottlenecked viruses. Cells were inoculated with vesicular stomatitis virus (VSV), and individual cells were transferred to separate culture wells with a micromanipulator. After overnight incubation, single, isolated plaques (viral progeny) from the supernatant were picked for massive parallel sequencing. The viral stock was subject to ultra-deep sequencing to detect the polymorphisms present in the inoculum (parental sequence variants). (**B**) The distribution of the number of non-parental single nucleotide polymorphisms (SNPs) found in the 7–10 plaques derived from each cell. (**C**) Distribution of the number of plaques derived from the same cell that contained a given non-parental variant. (**D**) Spectrum of nucleotide substitutions found after single-cell bottlenecks. (**E**) Correlation between the abundance of each type of substitution in single-cell-derived plaques and natural isolates. All panels adapted with permission from [12]. Copyright 2015, Elsevier.

The genomic mutation of pathogens during infection can be also detected directly. The sequencing of transcriptome and viral genes in single infected cells showed that IAV is highly prone to mutation during infection [11]. Detected mutations can cause consequences include viral polymerase malfunction and failure to express the interferon (IFN) antagonist protein, which is correlated to heterogeneous immune activation among infected cells [11]. The sequencing of 881 plaques from 90 VSV-infected cells detected 36 parental single nucleotide polymorphism (SNP) and 496 SNP generated during infection (Figure 1A–E) [12]. Although extremely low multiplicity of infection (MOI) was adopted, resulting in 85% of the cells statistically infected with only one PFU, 56% contained more than one parental variant, indicating that pre-existing differences in viral genomes can be spread within the same infectious unit, in this case, the host cell population. Moreover, by measuring the viral titers produced by each infected cell, a significant correlation was found between the number of mutations in the viral progeny and the log yield of the initially infected cell.

Genomic variability also widely exists among bacteria populations. Fluorescence labeling enables the quantification of bacterial growth in single host cells [13,14,15], and by correlating the heterogenous growth with host response, it was found that the *Salmonella* population exhibits different induction levels of the PhoP/Q two-component system, which modulates lipopolysaccharides (LPS) on the surface of individual bacteria [14].

### 2.2. Host Cell Heterogeneity

To understand the pathophysiology of infectious diseases, it is important to study the identities of targeted cells. Mounting evidence has shown that even under identical conditions, individual host cells manifest differential susceptibility and responses to infection in a population. How does this preference arise? Do they share similar features that might be reasons for their susceptibility of infection? How do the states of infected cells affect pathogen replication and infection outcome? Furthermore, how are host cells’ phenotypes influenced by infection individually and temporally? Answers to these questions are critical for the identification of target cells and individuals of novel pathogens, as well as for the understanding of infection pathophysiology.

Analysis of cells exposed to pathogens at single-cell resolution requires, first and foremost, strategies to distinguish infected cells from uninfected ones. Pathogen-specific proteins, such as viral glycoproteins embedded in the cell membrane, or intracellular proteins such as viral capsid or polymerases, as well as pathogen nucleic acids, including genomic DNA/RNA and transcripts, can serve this purpose. These microbial elements can be labeled with specific antibodies or oligonucleotide probes for detection and quantification. Alternatively, pathogen nucleic acids can be directly captured in deep sequencing. By combining tools for pathogen identification with host cell phenotyping assays, infected cells can be profiled at the single-cell level.

Xin et al. investigated the effects of host cell heterogeneity on both acute and persistent infection by foot-and-mouth disease virus (FMDV) [16]. By sorting single infected cells with FACS based on cellular parameters, and quantifying viral genome replication with RT-PCR, they showed that the host cell size and inclusion numbers affected FMDV infection. Cells with larger size and more inclusions contained more viral RNA copies and viral protein and yielded a higher proportion of infectious virions, which is likely due to favorable virus absorption. Additionally, the viral titer was 10- to 100-fold higher in cells in G2/M than those in other cell cycles, suggesting that cells in the G2/M phase were more favorable to viral infection or for viral replication. Such findings have also been reported for other viruses [9,17,18], revealing a general effect of heterogeneous cell cycle status in a population on virus infection.

Golumbeanu et al. demonstrated host cell heterogeneity using scRNA-seq: they showed that latently HIV-infected primary CD4^+^ T cells are transcriptionally heterogeneous and can be separated in two main cell clusters [19]. Their distinct transcriptional profiles correlate with the susceptibility to act upon stimulation and reactivate HIV expression. In particular, 134 genes were identified as differentially expressed, involving processes related to the metabolism of RNA and protein, electron transport, RNA splicing, and translational regulation. The findings based on in vitro infected cells were further confirmed on CD4^+^ T cells isolated from HIV-infected individuals. Similarly, enabled by scRNA-seq and immunohistochemistry, several candidate Zika virus (ZIKV) entry receptors were examined in the human developing cerebral cortex and developing retina, and *AXL* was identified to show particularly high transcript and expression levels [20,21].

scRNA-seq can also be used to identify potential target cells of novel pathogens and facilitate the understanding of disease pathogenesis and treatment. The spike protein of the virus SARS-CoV-2, the pathogen responsible for the COVID-19 pandemic, binds with the human angiotensin-converting enzyme 2 (ACE2) [22,23]. This binding, together with a host protease type II transmembrane serine protease TMPRSS2, facilitates viral entry [22,23]. By analyzing the existing human scRNA-seq data, it was identified that lung type II pneumocytes, ileal absorptive enterocytes, and nasal goblet secretory cells co-express *ACE2* and *TMPRSS2*, which suggests that they might be the putative targets of SARS-CoV-2 [24].

In the preparation of scRNA-seq library, standard poly-T oligonucleotide (oligo-dT) is commonly used to capture mRNA from single cells, which can also capture polyadenylated viral transcripts from DNA virus or negative-sense single stranded RNA virus. A simultaneous analysis of host transcriptome profiles and viral DNA/RNA offers information on the presence of the studied pathogen and its activities and allows a more accurate characterization on the dynamics of host–pathogen interactions.

**Figure 2:**
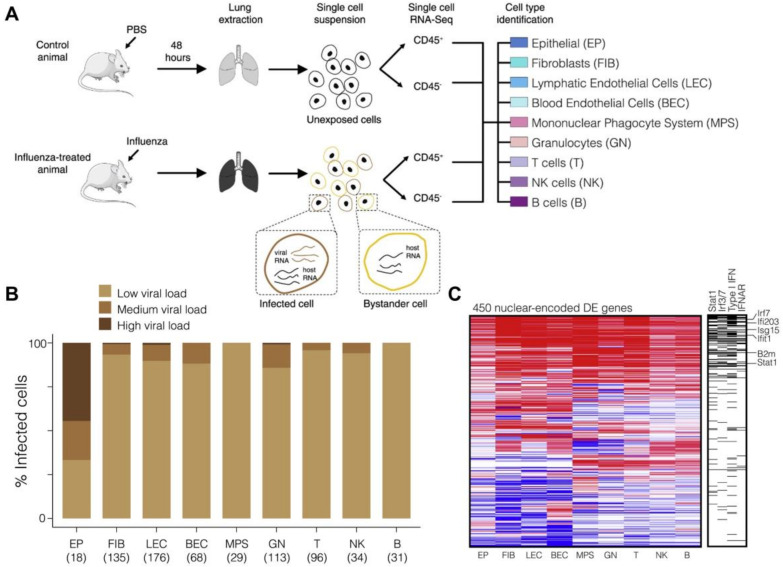
Single-cell analysis of influenza-infected mice lung tissues demonstrated heterogeneous virus load and gene expression activation in infected cells. (**A**) Schematic illustration of the experimental workflow. Immune and non-immune single cells were isolated from the whole lung of control and influenza-treated mice for massively parallel single-cell RNA sequencing. Host and the viral mRNA were simultaneously measured, allowing the identification of infected as opposed to bystander cells, the quantification of intracellular viral load, and the profiling of transcriptomes. Nine cell types were distinguished based on their transcriptional identities (**B**) The single-cell heterogeneity of intracellular viral load during influenza infection. Percentages of low (yellow), medium (light brown), and high (dark brown) viral-load states (y axis) within the population of infected cells are shown for each of the nine cell types (x axis; total numbers of infected cells are indicated). (**C**) Host genetic responses across all cell types. Differential expression in influenza-treated and control mice (color bar) of nuclear-encoded genes (rows) across the nine major cell types (columns). Right column indicates membership in four type I interferon (IFN)-related categories. All panels adapted with permission from [26]. Copyright 2018, Elsevier.

Wyler et al. profiled the transcriptome of single human primary fibroblasts before and at several time points post-infection with herpes simplex virus-1 (HSV-1), and they described a temporal order of viral gene expression at the early infection stage [25]. More importantly, by simultaneously profiling the host and viral mRNA, they identified that transcription factor NRF2 is related to the resistance to HSV infection. The finding was verified with the evidence that NRF2 agonists impaired virus production. Steuerman et al. performed scRNA-seq of cells from mice lung tissues obtained 2 days after influenza infection [26]. FACS was applied to sort immune and non-immune cells based on CD45 expression. Nine cell types were clustered (Figure 2A), and viral load was determined by the proportion of reads aligned to influenza virus gene segments, with higher than 0.05% considered infected. The authors found that viral infection can be detected in all cell types, and the percentage ranges from 62% in epithelial cells to 22% in T cells. However, the high variability of viral load was only observed among epithelial cells, while the majority of infected cells of other cell types showed to have low viral load (less than 0.5%) (Figure 2B).

For positive sense RNA virus whose transcripts lack polyadenylation and cannot be captured by oligo-dT, a reverse complementary DNA oligo probe to the positive-strand viral RNA was employed. Zanini et al. described this method and correlated gene expression with virus level in the same cell to study the infection of dengue virus (DENV) and Zika virus (ZIKV). They identified several cellular functions involved in DENV and ZIKV replication, including ER translocation, N-linked glycosylation, and intracellular membrane trafficking [27]. Interestingly, by contrasting the transcriptional dynamics in DENV versus ZIKV-infected cells, differences were spotted in the specificity of these cellular factors, with a few genes playing opposite roles in the two infections. Genes in favor of DENV (such as *RPL31*, *TRAM1*, and *TMED2*) and against DENV infection (such as *ID2* and *CTNNB1*) was also validated with gain/loss-of-function experiments.

Analysis methods have been advancing for the detection of genetic variant-based scRNA-seq data [28,29,30]. They could contribute, in the study of infectious diseases, to the characterization of temporal changes in viral mutational prevalence [31]. Moreover, viral mutation can be correlated with host gene expression status at the single-cell level to further investigate their potential mutual effect on one another throughout the course of infection and reveal the dynamic host responses and pathogen adaptations in the progression of infection [32].

In spite of the above-mentioned examples characterizing virus presence with scRNA-seq, it is worth noticing that viral mRNA or genome occurrence is not necessarily equivalent to viral progeny, due to reasons such as missing essential genes caused by mutations. Experimental techniques enabling the joint analysis of host transcriptional responses and viral titers will be needed to reveal the underlying mechanisms of virus production levels and host cell heterogeneity. Another challenge of analyzing viral RNA data is distinguishing infected cells with intracellular viral transcription from uninfected cells acquiring exogenous viral RNA. Combining single-cell transcriptomics data with flow cytometry or mass cytometry by time-of-flight (CyTOF) to measure the intracellular viral protein may help overcome this issue.

### 2.3. Host Immune Responses in Infection

Immune responses activated by infection, since it is the innate immune responses that are primarily initiated in infected cells, or adaptive immune responses by lymphocytes carrying specific roles, are dynamic and complex, and they often happen in specific tissue microenvironments. Heterogeneity in immune responses is also a long-recognized phenomenon. For instance, the activation of antiviral responses in dendritic cells (DCs) by bacterial LPS starts with a small fraction of cells initiating the reaction, followed by the response by the rest of the population via paracrine responses [33]. Technologies that enable the simultaneous measurement of multiple parameters facilitate high-resolution characterization of transcripts and protein at the single-cell level and boost our understanding of how host immune responses are initiated and orchestrated against infection. Although pathogens usually dominate the war with host immune responses, hence the prevalence of infectious diseases, in-depth understanding of the interplay provides valuable information for the design of strategies to fight against infectious diseases. In this section, we cover the single-cell characterization of both innate immune responses from infected cells and adaptive immune responses activated in infected units.

Type I interferon (IFN), a key cytokine in innate immunity, orchestrates the first line of host defense against infection. Its production is initiated upon host cells sensing pathogen-specific molecules, and it turns on the antiviral state of host cells by activating the transcription of hundreds of IFN-stimulated genes (ISGs), some of which are crucial for coordinating adaptive immune responses. Many studies have shown a large variability of IFN expression among infected cells. In the case of influenza virus infection, this can be partially explained by the high mutation rate during replication, revealed by sequencing viral genes in single infected IFN reporter cells [11]. However, such viability was also found to exist in infected cells expressing unmutated copies of all viral genes, which might be a result of the stochastic nature of immune activation irrelevant to viral genotypes [11].

In another study, PBMCs from patients with latent tuberculosis infection (LTBI) or active tuberculosis (TB), and from healthy individuals were analyzed with scRNA-seq [34]. T cells, B cells, and myeloid cells were distinguished, and 29 subsets were clustered. The novel finding in this work is the consistent depletion of one natural killer (NK) cell subset from healthy individual samples to samples from LTBI and TB, which was also validated by flow cytometry. The discovered NK cell subset could potentially serve as a biomarker for distinguishing TB from LTBI patients, which is valuable for predicting disease outcome and developing treatment strategies. By analyzing scRNA-seq data of PBMCs derived from individuals before and at multiple time points after virus detection, Kazer et al. investigated the dynamics of immune responses during acute HIV infection [35]. After identifying well-established cell types and subsets in PBMCs, the authors examined how each cell type varies in phenotype during the course of infection. Genes involved in cell-type specific activities, including monocyte antiviral activity, dendritic cell activation, naïve CD4^+^ T cell differentiation, and NK trafficking manifested similar changes with plasma virus levels: peaking closer to detection and gradually descending with time.

Phenotypic variations in bacteria populations were shown to influence host cell responses. Avraham et al. investigated macrophage responses against *Salmonella* infection with fluorescent reporter-expressing bacteria and scRNA-seq on host cells [14]. Transcriptional profiling revealed the bimodal activation of type I IFN responses in infected cells, and this was correlated with the level of induction of the bacterial PhoP/Q two-component system. Macrophages that engulfed the bacterium with a high level of induction of PhoP/Q displayed high levels of the type I IFN response, which was presumably due to the surface LPS level related to PhoP/Q induction. With a similar setup, Saliba et al. studied the *Salmonella* proliferation rate heterogeneity in infected macrophages [13]. The varied growth rate of bacteria, indicated by fluorescent expression by engineered *Salmonella* in single host cells, influenced the polarization of macrophages. Those bearing nongrowing *Salmonella* manifested proinflammatory M1 macrophages markers, similar with bystander cells, which were exposed to pathogens but not infected. In comparison, cells containing fast-growing Salmonella turned to anti-inflammatory, M2-like state, showing that bacteria can reprogram host cell activities for the benefit of their survival.

The above-mentioned strategy to simultaneously profile host cell transcriptome and viral RNA also plays an important role in characterizing immune responses against infection by identifying infected immune cells and analyzing the transcriptomes simultaneously. For instance, it was applied to study the heterogeneous innate immune activation during infection by West Nile virus (WNV) [17]. High variability was revealed for both viral RNA abundance and IFN and ISGs expression. Interestingly, the expression of some ISGs, with *Tnfsf10*, *Ifi44l*, and *Mx1* being the most prominent examples, was found to be negatively correlated with viral RNA abundance, which could be a direction for future studies on WNV-mediated immune suppression in infected cells. Similarly, Zanini et al. studied the molecular signatures indicating the development of severe dengue (SD) infection by analyzing single PBMCs derived from patients [36]. FACS was employed to sort PBMCs into different cell types (T cells, B cells, NK cells, DCs, monocytes), and then scRNA-seq was performed. The majority of viral RNA-containing cells in the blood of patients who progressed to SD were naïve immunoglobulin M (IgM) B cells expressing CD69 and CXCR4 receptors, as well as monocytes. Transcriptomic profiling data indicated that various IFN regulated genes, especially MX2 in naive B cells and CD163 in CD14^+^CD16^+^ monocytes, were upregulated prior to progression to SD.

Comparison of the single-cell transcriptomes of lung tissue from health and influenza-infected mice revealed that 101 genes, among which the majority are ISGs and targets of antiviral transcription factors, were consistently upregulated among all nine identified infected cell types, including both immune and non-immune cells [26]. This finding suggested that antiviral innate responses against influenza infection generically exist (Figure 2C). Moreover, by contrasting the expression profiles among infected, bystander, and unexposed cells, it was shown that the non-specific IFN gene module is a result of extracellular exposure and responses of environmental signals.

While single-cell transcriptomics analysis provides an unbiased determination on host cell states, proteomics analysis offers direct characterizations of proteins expressed upon pathogen activation. Going beyond traditional flow cytometry, mass spectrometry, or cytometry by time-of-flight (CyTOF) offers vastly increased numbers of parameters that can be investigated simultaneously, exponentially increasing the depth of the dataset collected. For instance, to investigate the effect of a precedent dengue virus infection on the outcome of subsequent Zika infections, PBMCs derived from patients with either acute dengue infection or health individuals were incubated with dengue virus or Zika virus, and the treated PBMCs were assessed by multiparameter CyTOF [37]. CyTOF in this study allowed the simultaneous detection of changes in the frequency of immune cell subpopulations and quantification of functional activation markers and cytokines in distinct cell subsets. While secondary infection with dengue virus led to increases of CD4+ T cells and T cell subsets, which are involved in adaptive immunity, secondary infection with Zika virus induced the upregulation of several functional markers including IFNγ and macrophage inflammatory protein-1β (MIP-1β) in NK cells, DCs, and monocytes, indicating an intact innate immunity against Zika virus in the cases of possible concurrent dengue infection. Hamlin et al. compared two DENV serotypes (DENV-2 and DENV-4) in their infection in human DCs using CyTOF, which allowed simultaneous analysis on DENV replication, DC activation, cytokine production, and apoptosis [38]. The tracking of intracellular DENV proteins and extracellular viral particles showed different replication kinetics yet similar peak viral titers by these two serotypes, as well as the percentage of infected DCs. Moreover, DENV-4 infection was found to induce a higher expression of CD80, CD40, and greater production of tumor necrosis factor-α (TNFα) and interleukin-1β (IL-1β), compared to DENV-2 infection. Additionally, bystander cells, which were identified by the absence of intracellular viral proteins, were identified to produce less TNFα and IL-1β, but show more activation of interferon-inducible protein-1 (IP-1), which is a member of ISGs.

Besides CyTOF, host cell secretomes can also be measured with customized miniatured systems, and the level of multiplexing and flexibility of sample handling is often improved. For instance, Lu et al. showed the co-detection of 42 secreted proteins from immune effector cells stimulated with LPS [39]. In a similar setup, Chen et al. performed a longitudinal tracking of secreted proteins from single macrophages in response to LPS treatment [40]. These studies provide valuable insights into the dynamic and comprehensive responses to pathogen over time. Notably, such methods require microfabrication tools and skills, which is not always available and thus hinder their accessibility, compared with flow cytometery and CyTOF.

Epigenetic profiling at the single-cell level is also important, especially for elucidating the influence of host immune responses in chronic infection. The Assay for Transposase-Accessible Chromatin with high throughput sequencing (ATAC-Seq) utilizes Tn5 transposase to insert sequencing adapters into regions of open chromatin, in order to study genome-wide chromatin accessibility. Buggert et al. applied ATAC-seq and established the epigenetic signatures of HIV-specific memory C8^+^ T cells resident in lymphoid tissue [41]. Yao et al. used chromatin immunoprecipitation followed by high-throughput sequencing (ChIP-Seq) to examine the histone modification of progenitor-like CD8^+^ T cells from mice chronically infected with lymphocytic choriomeningitis virus (LCMV) [42]. They found that progenitor-like CD8^+^ T cells showed distinct epigenomic features compared with memory precursor cells, exhibiting more abundant active histone markers (H3K37ac modification) at genes co-expressed with *Tox*, which encodes the thymocyte selection-associated high mobility group box protein TOX. This might promote the long-term persistence of virus-specific CD8^+^ T cells during chronic infection.

In some cases, deep sequencing can be implemented together with other single-cell technologies for a comprehensive and systematic profiling of immune responses against infection. For instance, Michlmayr et al. performed 37-plex CyTOF on peripheral blood mononuclear cells (PMBCs), RNA seq on whole blood, and serum cytokine measurement of blood samples from patients with chikungunya virus (CHIKV) infection [43]. Moreover, samples collected at acute and convalescent phases were compared to study the disease progression. Such multidimensional analysis allows the large-scale, unbiased characterization of gene expression, cytokine/chemokine secretion, and cell subpopulation changes in response to infection. One important result of this study is revealing monocyte-centric immune response against CHIKV, with the frequency of two subsets both related to antibody titers and antiviral cytokine secretion. In addition, significant viral protein expression was found in two B cell subpopulations.

While multiple assays can be done on the same bulk sample to obtain different data parameters (e.g., transcriptomic, proteomic), such datasets are not able to correlate the data parameters at the resolution of a single cell. Newer advances allow the simultaneous collection of multiple types of parameters for the same cell. For instance, Cellular Indexing of Transcriptomes and Epitopes by Sequencing (CITE-seq) and RNA Expression and Protein Sequencing (REAP-seq) are techniques for the simultaneous collection of transcriptomic and high-dimensional information on specified proteomic targets. By using antibodies tagged with unique nucleotide sequences, the subsequent transcriptomic sequencing simultaneously sequences these tags to allow the quantification of the antibody targets. Corresponding transcriptomic and proteomic data at the single-cell level allows the opportunity to study the role of post-translational gene regulation in the immune response. The increased dimensionality of the information obtained may also allow more accurate machine learning to identify signatures of healthy or dysfunctional immune responses. For instance, using CITE-seq, Kotliarov et al. were able to identify a common signature of activation in a plasmacytoid dendritic cell-type I interferon/B lymphocyte network that was associated both with flares of systemic lupus erythematosus (SLE) and influenza vaccination response level [44].

## 3. Therapeutics Discovery

### 3.1. Single-Cell Technology in Therapeutics Discovery and Clinical Application

As noted above, the ability to study biological processes at the single-cell level gives an unprecedented to attribute bulk phenotypes in immunology and host–pathogen interaction to specific cell subpopulations, including rare cell populations, in a relatively unbiased fashion. Apart from basic science discovery, how do these insights affect clinical practice in infectious disease? Biomarker discovery is one obvious area of impact—the molecular differences found to underpin broader disease phenotypes can be used to diagnose or even predict disease. In particular, diagnosis is a notable problem in infectious disease, where identification of the causative pathogen can take days to weeks for culture-based systems, which may delay appropriate, targeted treatment [45]. Apart from biomarker discovery, single-cell technology is also revolutionizing the discovery of vaccines and therapeutics, which will be elaborated upon in the sections below. Other clinical uses of single-cell technology may require an increased uptake of such technologies within the hospital setting. For instance, one potential area of impact is antimicrobial resistance. The bulk genotype or phenotype of a pathogen population may not accurately identify its ability to become resistant to antimicrobials, since antimicrobial resistance can involve the selection of a previously rare, resistant population. Should single-cell technology become routinely used in hospitals, the increased resolution could enables the identification of such rare populations, which can inform the choice of antimicrobials prescribed. To generalize, this similarly applies to any disease phenotype that can be triggered by a rare host or pathogen cell population. The complexity of current single-cell technologies hinders their implementation in the clinic, and in the section titled Diagnostics, we highlight various steps that have been taken toward simplifying single-cell technology platforms to allow their clinical use.

### 3.2. Vaccine Development

The first step of the vaccine development pipeline would be to identify a promising disease antigen, which could be in the form of a recombinant protein or inactivated/attenuated virus. Unlike traditional vaccinology where vaccines were generated via pathogen growth and inactivation, the reverse vaccinology approach relies on predicting antigen features that are likely to trigger protective functions and engineering the antigen accordingly [46]. To predict these antigen features, two main approaches have been used: via whole genome sequencing and more recently, identifying and mapping the structural epitopes of neutralizing antibodies using the methods discussed later in this review [47].

After identifying a vaccine candidate, the next step would be to verify its efficacy. This efficacy is quantified based on its ability to bring about a set of specific immune responses which are specifically linked with protective functions, which are known as Correlates of Protection (CoPs) [48]. It is important to identify the CoPs for each vaccine for multiple reasons, including the following: (1) to understand the mechanisms of vaccine protection for improvement of vaccines, (2) to understand the mechanisms of vaccine protection for improvement of vaccines, (3) to determine the consistency of the vaccines produced, (4) to evaluate the levels of protection to patients before and after treatment, and (5) for the licensure of said vaccine [49]. Historically, most of the CoPs in commercial vaccines typically involve quantifying the titer of neutralizing antibody produced by antigen-specific memory B cells. In the past decade, better understanding of the in vivo vaccine response has led researchers to identify several relevant memory T-cell responses as CoPs, and these T-cell responses are usually quantified by measuring the expressed cytokines via techniques such as ELISpot, flow cytometry, and ELISA [50].

However, it remains difficult to define vaccine CoPs for a number of diseases. These include those diseases that cannot yet be eliminated by vaccine or infection-elicited immune responses (e.g., HIV-1 infection, tuberculosis), since a suitable end point of protection is not attainable. They also include those diseases for which vaccines do not yet exist but vaccine CoPs may be expected to differ from infection-related CoPs, including diseases for which natural clearance occurs via the innate immune response or early adaptive immune response (e.g., COVID-19). Even when immune parameters that correlate with disease risk are found, the causative mechanism of immune protection, or mechanistic CoP, may remain elusive if multiple immune parameters are elicited in parallel by a protective response. As seen in the excellent review by Plotkin [51], the CoPs may not always be as obvious or limited to humoral immunity, and since vaccines typically elicit multiple immune responses. This is especially true for the case of vaccines against complex pathogens such as HIV and malaria, where the resultant network of immune responses may not always be easily identifiable.

Single-cell approaches may define a greater space of immune parameters to be explored as CoPs. Furthermore, the increased breadth of data that can be obtained from a single sample is useful in increasing the number of hypotheses that can be probed, especially in longitudinal analyses, which are most useful for mechanistic immune studies but where the sample volume is often limited.

Furthermore, using a systems vaccinology approach via omics technology, researchers have begun to uncover these potential CoPs early in the vaccine development process [52]. In one of the earliest proof-of-concepts, Querec et al. successfully identified a CoP for vaccine efficacy on humans vaccinated against yellow fever. A gene marker present in CD8+ T cells which could predict for protection was discovered by using a multivariate analysis of the immune response via a combination of flow cytometry and microarray techniques [53].

With the rapid developments in single-cell omics technology, a deeper understanding of vaccine response can be obtained through an even more detailed mapping of the interactions between the various immune cell populations at the single-cell level, as well as identify the causes of heterogeneous vaccine response in individual immune cells [54]. This could be seen from the recent work by Waickman et al. [55] where a dengue vaccine elicited a highly polyclonal repertoire of CD8+ T cells that was identified using scRNA-seq. Combined with transcriptional analysis of the CD8+ T cells, the authors established a set of metabolic markers that could be potential CoPs for vaccine efficacy evaluation. Combining the simultaneous analysis of single-cell transcriptomic and TCR sequence data, Tu et al. identified preferential transcriptional phenotypes among subsets of expanded TCR clonotypes. This is a strategy that may be highly valuable in assessing the functionality of T cells and their correlation to protection in vaccine responses [56].

### 3.3. Antibody Discovery

Antibodies are widely used in therapeutics and diagnostics due to their high specificity and generally low toxicity. Antibodies are capable of mediating protective functions against infectious diseases, including pathogen neutralization, antibody-mediated phagocytosis, antibody-mediated cellular cytotoxicity, and complement-dependent cytotoxicity. Antibody-containing sera remains in use for diseases where there are no other therapeutic options, including for viruses such as Hepatitis A or B, Rabies, Vaccinia, SARS-CoV-2 at the point of writing, and for toxins (e.g., snake venom). However, there are limitations to this approach: serum therapy from animal sources causes a risk of serum sickness due to immune reaction against animal protein, while pooled hyperimmune sera from humans is difficult to collect and standardize. Instead, the appropriate B cell clone that secretes antibody with protective activity can be isolated, and its antibody sequence can be obtained and expressed in culture to obtain monoclonal antibodies as therapeutics. Similarly, in diagnostics, monoclonal antibodies provide the specific recognition of pathogen antigens that allow the rapid diagnosis of infection.

In order to identify the correct B cell clone from thousands or millions of B cells, its antigen specificity and/or protective activity must be interrogated. This is classically done by cell immortalization (such as by hybridoma production or Epstein–Barr virus infection to generate B lymphoblastoid cell lines), followed by single-cell plating and expansion to obtain sufficient antibody from a single clone, and then the well-based screening of the antibody-containing cell supernatants. However, these techniques are low in throughput and efficiency, losing more than 99% of potential cells [57,58] for hybridomas, and 70–99% of potential cells for B lymphoblastoid cell lines [59,60]. Moreover, there remains a bottleneck in throughput at the subsequent stage of subcloning and screening the resulting clones to determine which clones are antigen-specific and functional for the desired purpose—even large experiments are limited to screening several thousand cells [61,62], or up to 100,000 cells for robot-assisted operations [63], whereas a single 30 mL human blood draw contains an order of magnitude more (approximately 900,000) candidate CD27+ IgD- class-switched memory B cells [64].

More recently, techniques that avoid the need for cell expansion have been developed—these speeds up the life cycle for monoclonal antibody discovery. Primary B cells expressing antigen-specific B cell receptors (BCRs) are labeled using fluorescent antigens, allowing flow cytometry-based single-cell sorting to isolate these antigen-specific B cells [65]. This technique is useful especially for the interrogation of memory B cells, which express the BCR on their surface. The interrogation of plasmablasts and plasma cells, which secrete antibodies but have low or no surface expression of the BCR, require other procedures such as the formation of an Ig capture matrix on the B cells [66], or alternative methods of screening that allow the physical separation of single cells such as droplets [67], nanowells [68,69,70,71], or microcapillaries [72]. Following the isolation of the desired B cells, they are lysed and their RNA is interrogated to recover the antibody heavy and light chain genes. With these techniques, both antigen interrogation and antibody gene recovery do not require large clonal cell populations, removing the need for inefficient and time-consuming cell expansion processes.

For the recovery of antibody genes, RT-PCR is commonly used, but recovery rates are typically low (<70% success rate for each pair of heavy and light chains) due to the large variability across the V gene families. Single-cell RNA-seq (Smart-seq2) is an alternative to RT-PCR, which results in improved recovery rates (>90%) [73]. BCR recovery can also be done in the same step as antigen-specific sorting via the use of DNA-barcoded antigens, such that both the antigen barcodes and BCR sequence are recovered simultaneously during single-cell NGS [74]. This has been used to successfully isolate broadly neutralizing HIV-1-specific antibodies and influenza-specific antibodies simultaneously from a single sample, although the resulting antibody candidates had variable neutralization functions, which required subsequent in vitro confirmation.

Another method for monoclonal antibody discovery is the use of phage display libraries, where phages expressing antibody genes are selected for using an antigen-coated surface in an iterative process of biopanning [75]. This has been a fast and effective method for monoclonal antibody discovery. The main limitation of phage library display is the random, largely non-native pairing of VH and VL genes, which may cause problems in subsequent antibody expression and production, and it may also have a higher likelihood of triggering anti-idiotypic allergic responses. More recently, a single-cell emulsion technique has been used for the interrogation of antigen specificity and high-throughput sequencing, allowing the interrogation of a yeast library utilizing natively paired human antibody repertoires [76]. Using it, rare broadly neutralizing antibodies against HIV-1 could be identified, albeit with the correct antigen required for identifying the desired B cell clones.

Antigen binding is the most common form of screening for monoclonal antibodies due to its compatibility with high-throughput methods including flow cytometry, biopanning, and nanowell-based ELISA. However, antigen binding may not correlate with functional activity against the intended target. For example, this may occur if the protein antigen used does not accurately mimic the native form of the antigen; monoclonal antibodies generated against the protein antigen may not be active against the native target [77,78,79,80]. Another example would be if functional activity requires binding in a specific orientation, such as virus neutralization requiring the monoclonal antibody to disrupt the receptor binding site [81,82]. Assays for monoclonal antibody function include assays for virus neutralization, opsonophagocytosis, antibody-dependent cellular cytotoxicity, and receptor agonism/antagonism [83]. Currently, these assays are typically done in bulk with relatively low throughput, creating a bottleneck in monoclonal antibody screening.

Microfluidic technologies, such as water-in-oil emulsions or nanowells, are being developed to increase the throughput of such assays. For instance, a high-throughput screen for enzyme antagonism using a droplet-based assay has been reported [84]. Using water-in-oil microdroplets, El Debs et al. co-encapsulated single hybridoma cells with an enzyme (ACE-1) and an enzyme substrate that emits a fluorescence signal upon enzyme hydrolysis, and they were able to sort out hybridomas secreting ACE-1-inhibiting antibodies through fluorescence-activated droplet sorting. Another group has recently also reported assays that are capable of assaying cellular internalization, opsonization, and the functional modulation of cellular signaling pathways [67], and several companies have also reported proprietary platforms that may be able to carry out some other functional assays [85]. However, the specificity and sensitivity of these assays have not been reported. The ability to immobilize single cells in nanowells allows repeated longitudinal profiling, which is a property that was utilized by Story et al. to obtain antibody–antigen binding curves that can classify related populations of B cells [71].

### 3.4. Antibiotic Discovery and Antimicrobial Resistance

The characterization of diverse bacterial populations, including microbiome studies, has traditionally been done at the bulk level. For instance, the selection of particular organisms out of a diverse population has been done by the plating and amplification of single colonies. However, this method is limited in throughput. The enhancement of throughput can be done via miniaturization—for instance, one study isolated antibiotic-resistant *E. coli* mutants by encapsulating and culturing single bacteria in nanoliter-scale droplets containing the antibiotic [86]. This approach can be applied to accelerate the identification of targets acted upon by antibiotics of unknown mechanisms.

Apart from being limited in throughput, traditional microbial selection systems also require the ability to culture the microorganism of interest in vitro. However, it is estimated that the bulk of microorganisms cannot be cultured and expanded in typical cell culture media [87]. One potential solution is to use microfluidic devices to physically separate and phenotype individual bacteria while immersing them in media derived from their natural environment. This method was adopted to identify a new antibiotic, teixobactin, from a previously unculturable β-proteobacteria belonging to a group of Gram-negative organisms not previously known to produce antibiotics [88].

## 4. Diagnostics

### 4.1. Disease Monitoring and Clinical Diagnostics

In the clinical setting, single-cell analysis techniques are currently rarely routinely used in infectious disease diagnostics and monitoring. It is still impractical to apply most of the other conventional single-cell analysis techniques for diagnostic applications due to the associated high costs, long workflow durations, and high degree of technical expertise required. One notable exception would be flow cytometry, where aside from its high initial equipment cost, its fast turnaround times, high sensitivity, and ease of operation make it a staple tool in clinical institutions worldwide [89]. Flow cytometry is mainly used to perform the immunophenotyping of blood cells against various disease-specific biomarkers [90,91,92]. The most prominent example would be in the routine monitoring of human immunodeficiency virus (HIV) progression by counting the number of CD4^+^ T cells in a patient’s blood sample [91].

The other single-cell technique that has seen some use in diagnostics against pathogens would be fluorescence in situ hybridization (FISH). As a diagnostic tool, FISH has numerous advantages that include low cost and complexity; its rapid turnaround time allows the diagnosis of fastidious bacteria and the ability to distinguish between mixed populations of pathogens at a single-cell resolution [93]. While FISH has been successfully used for the direct identification of panels of pathogens from blood samples [94,95], its reliance on image analysis as the readout limits the throughput of this technique, and the results are subject to user-to-user variation and bias [96]. To resolve these issues, a variant of the technique, FISH-flow, was developed. FISH-flow combines FISH with flow cytometry to achieve higher throughputs as well as automates the signal readout through the cytometric system [97], and it has been used to detect HIV reservoirs in T cells [98] as well as bacteria from blood [99].

### 4.2. Toward Point-of-Care Applications

**Figure 3:**
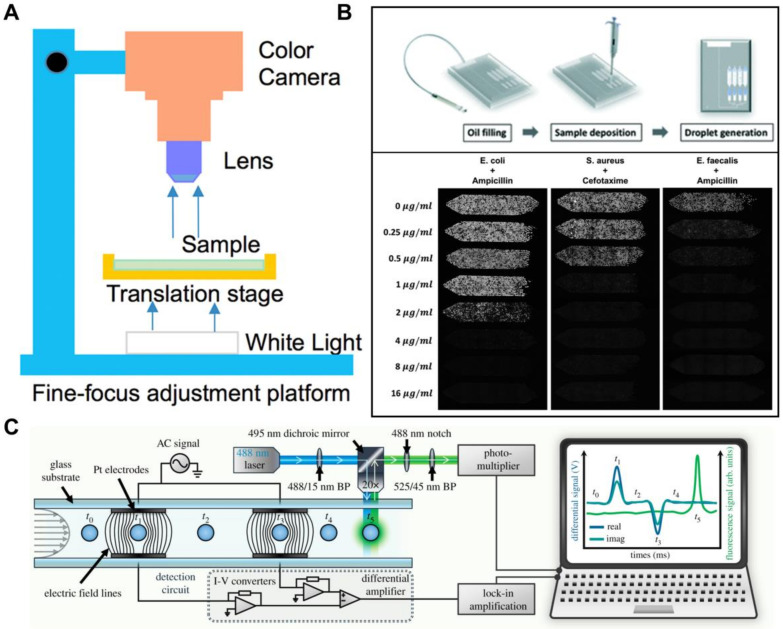
Single-cell platforms for infectious disease diagnostics. (**A**) Portable image cytometer capable of performing the automated counting of cells containing malaria parasite. Image reproduced from reference [102] under a Creative Commons License. (**B**) Pump-free droplet emulsion generation system that is capable of performing antimicrobial-susceptibility testing (AST) of different species of bacteria with a turnaround time of ≈5 h. Image reproduced with permission from reference [117]. Copyright 2020 Royal Society of Chemistry. (**C**) Microfluidic impedance cytometry is able to differentiate between healthy and malaria-infected red blood cells at a single-cell resolution based on the difference in electrical impedance measured across two electrodes. Image reproduced from [122] under a Creative Commons License.

While the ability to identify biomarkers at a single-cell resolution is certainly invaluable in the fight against infectious diseases, current flow cytometer systems are typically bulky and expensive, thereby limiting their use in a laboratory setting [100]. Fortunately, advancements in microfluidics and low-cost electronics have given rise to the development of portable platforms that can perform single-cell analysis in a point-of-care (POC) setting. Recent examples of portable cytometric systems that are relevant to infectious disease diagnosis include a miniaturized modular Coulter counter capable of label-free detection and the differentiation of particles of varying sizes [101], a low-cost and portable image-based cytometer for the quantification of malaria-infected erythrocytes [102] (Figure 3A), and a portable miniaturized flow cytometer that is capable of multi-channel fluorescence interrogation of whole blood samples [103].

The portability of such cytometers could mean faster turnaround test timings through on-site diagnostics and disease monitoring, hence expediting clinical decisions and improving healthcare outcomes in general [104]. In addition, the portability of such microfluidic systems lends to other practical applications of flow cytometry, especially in pathogen detection in water and food sources. Particularly, diarrheal diseases (a leading cause of death for children under the ages of 5) are closely linked to the consumption of contaminated water sources and could be mitigated via regular, on-demand pathogenic testing of drinking water [105].

However, adapting current single-cell technologies into a portable format holds its own set of unique challenges. Most of the existing literature surrounding such technologies still report separate sample enrichment or staining steps prior to cell analysis [106,107,108]; such additional preparatory steps increase assay complexity, which may not be desirable in a POC setting [109]. While a gamut of existing microfluidic technology has already been established for sample purification as well as for reagent addition and mixing, integrating the various modules into a single platform is typically not a trivial process [110]. For single-cell technology to make the successful transition from the lab to the bedside, such practicalities must be considered and successfully implemented.

### 4.3. Digital Assays

Digital assays are a relatively recent assay format comprised of the following steps: (1) the discretization of a single initial larger sample volume into multiple smaller volumes (typically via microwell, microvalve, or droplet emulsion partitioning techniques [111]), and (2) performing the chemical or biological assay on each individual volume to obtain a quantifiable signal [112]. Due to the ability to individually assay a large number of cells at the single-cell level, the digital assay format has been widely employed in single-cell omics studies [113]. In the field of infectious disease research, while most of the applications of digital assays have been centered on answering fundamental questions relating to pathophysiology, there are other single-cell diagnostic applications that can benefit tremendously from such an assay format.

An example mentioned earlier in the review would be rapid antimicrobial-susceptibility testing (AST) to address the surge of antimicrobial-resistant infections worldwide as a result of the misuse of antimicrobials. Phenotypic AST, which involves the culture of the pathogen in the presence or absence of antibiotics, may help guide treatment options, but existing conventional assays have low sensitivity and require a long time of 12–48 h for cell regrowth to achieve measureable assay outcomes [114,115]. Higher-sensitivity single-cell digital assays that have been recently reported can obtain measurable signals without requiring cell regrowth and could be the answer to reducing AST turnaround times (Figure 3B) [116,117,118]. Another application of digital assays could be in quantifying viral reservoirs in patients at a single-cell resolution. In HIV eradication studies, latent reservoirs are reactivated using latency-reversing agents (LRAs) for subsequent inhibition via antiretroviral therapy [119]. The ability to isolate and individually assay the patients’ blood to obtain the distribution of reactivation states in the heterogenous cell population can give clinicians an idea of antiretroviral treatment efficacy in the future [120,121].

### 4.4. Label-Free Analysis

The development of label-free single-cell analysis techniques has been gaining considerable attention over the last decade with the advent of microfluidics because of their numerous advantages over their counterparts that require cell labeling. Some of the advantages include: (1) lower technical complexity and turnaround times in assay workflow because the preparatory step is omitted, (2) not requiring knowledge of cell biomarkers beforehand, making them suitable for assaying novel cell populations, and (3) by avoiding the use of labels that might affect the natural state of the cells, results might be more representative of actual in vivo cellular conditions [123]. Coupled with the precise fluid handling capabilities afforded by microfluidics systems, there is a burgeoning number of label-free single-cell analysis platforms that have been reported in recent years that are able to measure infection based on the inherent properties of the cells.

One prime example would be the identification of cells via their electrical properties, specifically electrical impedance. This impedance is derived from the change of voltage or current signal when single cells flow across a pair of miniaturized electrodes, and it has been shown to be able to differentiate between healthy and malaria-infected erythrocytes (Figure 3C) [122], as well as the viability and species of parasitic protozoa [124]. Another promising direction for label-free single-cell analysis is via measuring the inherent optical properties of cells. This has been shown in recent work such as the single-cell identification of parasites through their Raman spectra [125], the quantification of single-cell viral infection titer through Laser Force Cytology [126], and single bacteria detection via refractive index measurements [127]. Lastly, the mechanical and size properties of cells have also been exploited for identifying infected single cells. For example, using inertial microfluidics, white blood cells could be hydrodynamically isolated from lysed blood containing ring-stage malaria parasites as a result of the white blood cells’ larger sizes [128]. Other recent works that demonstrate potential applications for single-cell label-free infectious disease analysis includes cell identification via their acoustophoretic responses [129], as well as their deformability and hydrodynamic resistance [130].

Evidently, there is a host of promising label-free single-cell analysis technology that could be translated to clinical diagnostic applications. In the near future, these technologies would be useful complement POC applications where labeling steps in the assay workflow would increase the technical complexity and hinder the transition from the lab to bedside.

## 5. Considerations in Single-Cell Studies

Among the methods discussed, scRNA-seq is the primary tool for single-cell studies. In the following section, we briefly cover some important points that needs to be considered when designing and conducting such experiments. For a more in-depth coverage of this subject matter, the reader is invited to read other excellent reviews from Luecken [131], See [132], and Lähnemann [133].

### 5.1. Number of Cells and Sequencing Depth

As covered earlier in this review, the main applications of scRNA-seq in infectious disease study comprise of the following: (1) studying effect of host cell heterogeneity on infection, (2) identifying host immune responses, and (3) antibody discovery. However, the number of sequenced cells and depth of sequencing ultimately depend on the end goal of each experiment as well as the amount of financial resources at hand. The availability of a variety of commercial platforms for single-cell analysis with different throughput and sensitivity can provide users with different options to best suit the purpose of their studies [134].

For studies that involve identifying the cell types of a heterogeneous sample, a minimum of 50,000 reads per cell would be sufficient [135], while testing on a significantly large number of cells would ensure that rare subpopulations do not get missed out. One such application in infectious disease studies would be the systemic characterization of immune cell populations in response to an infection, wherein a large number of cells has to be screened in order to encompass the extensive diversity of B and T cells [136]. On the other hand, for studies which the main goal is to obtain a high resolution readout of the transcriptome for a small number of cells, 1,000,000 reads per cell would be a reasonable estimate [33].

### 5.2. Reproducibility and Reliability of Data

In typical bulk analysis, multiple biological and technical replicates can be performed in order to ensure the reproducibility of data. However, for single-cell experiments, particularly for scRNA-seq, there are two main issues to contend with. Firstly, measurements typically have high technical variability as replicate measurements cannot be performed on the same cell, which is lysed as part of the RNA extraction process. Secondly, the resulting single-cell data are typically noisy due to technical variations from the multitude of steps in scRNA-seq, as well as biological variation stemming from cell heterogeneity [137]. As such, great care has to be taken at each step of the scRNA-seq workflow (i.e., sample preparation, library preparation and sequencing, data analysis) to minimize such technical variability and batch effects.

One of the major sources of such variability arises from the initial sample preparation process. Regardless of how the cells are dissociated, purified, or enriched, cell expression is likely to change in response to the stress induced from these processes. To minimize such undesired changes which might affect downstream data analysis, the sample preparation protocol should be optimized iteratively for each cell type [138].

To reduce technical variability, one common method would be to spike = 0 in known quantities of synthetic RNA into the samples as controls to normalize read counts prior to data analysis [139]. A recent advancement in such RNA spike-in normalization methodology would be the BEARscc (Bayesian ERCC Assessment of Robustness of single-cell clusters), which generates simulated technical replicates based on the readout signal variation from spike-in measurements [140]. An alternative to RNA spike-ins would be the use of Unique Molecular Identifiers (UMIs) incorporated into the primers during reverse transcription, which essentially act as unique barcodes that allows the identification and subsequent tracking of transcribed mRNA. Then, the resulting data can be normalized against the UMI levels to account for amplification bias during the library generation step [141]. However, both RNA spike-in and UMI have their own set of limitations to consider; RNA spike-ins are unsuitable for protocols that utilize poly-T priming and template switching, and since they are typically used in large amounts relative to the endogenous RNA, they could potentially occupy a lot of reads. Protocols utilizing UMI need to ensure that library sequencing is sufficiently deep to cover all UMI transcripts; otherwise, there will be a risk of incorrectly quantifying the initial sample RNA [142].

Another source of error for scRNA-seq comes from batch effects, which are brought about by unavoidable variations between batches of experimental runs due to changes in environmental conditions, temperature, reagent lot, etc. In response, several computational methods have been developed to mitigate said batch effects from the scRNA-seq data. For example, one of the more commonly used batch-effect correction methods, ComBat, utilizes an empirical Bayesian framework that removes batch effects via a linear model, which factors in both the mean and variance of the scRNA-seq data [143]. For a more in-depth study on the comparative performance between the various batch-effect correction methods, we urge readers to consult a recent study by Tran et al. [144].

## 6. Future Outlook

### 6.1. General Limitations of Current Single-Cell Platforms

While single-cell platforms have indeed come a long way in the past two decades, the plethora of existing techniques still face a few general concerns that could present themselves as opportunities for development in the near future.

One of the inherent challenges in single-cell studies stems from the simple fact that the total amount of biological material present in a single cell is pretty limited and as a result, the resulting data are typically noisy from multiple biological and technical sources. Making sense of the data requires downstream data pre-processing and analysis, which are non-trivial components of the workflow that limits the accessibility of such studies to groups with the essential background. Additionally, with the increasing number and complexity of parameters at which single-cell assays are being performed, the curse of dimensionality is a pertinent problem that still requires further examination [133].

Another concern for single-cell platforms would be inter-experiment variability, as mentioned in the previous section. Single-cell technologies innately have high measurement sensitivity and thus are more susceptible to variations in results obtained from technical replicates, and methods to bioinformatically correct for such differences are required. Coupled with the fact that single-cell studies are typically expensive and therefore sample sizes are small, ensuring that results are comparable between each sample becomes an even more important issue.

Finally, the inability to maintain viable cells after analysis, particularly for high-throughput methods such as flow cytometry or scRNA-seq, gives rise to a couple of problems. Firstly, a majority of the conventional single-cell studies are limited to a single time point of study, following which the cells are discarded. Secondly, the irrecoverability of the cells makes it difficult to integrate back-to-back assays, which required measuring different parameters. As such, improvements in cell handling to improve cell viability would be invaluable in obtain multi-parametric datasets required for a more holistic understanding of cellular behavior.

### 6.2. Techniques on the Horizon

To date, the applications of single-cell technology have revolutionized our understanding of host–pathogen interactions. While many studies have focused on immune cells from the blood, the study of immune responses in the context of solid tissues or foci of infection (in both acute and chronic disease phases) is important to understand the local context of host–pathogen interaction. For this, techniques allowing the integration of spatial information with other single-cell technologies will be useful. For instance, imaging mass cytometry has been used to obtain quantitative information on 32 proteins at a spatial resolution of 1 µm [145]. This is done by systematically ablating a formalin-fixed tissue sample spatially line by line. The increased number of markers allows the fine distinction of cell subsets and activation states, providing valuable information on cellular roles in immune effector function or immunopathogenesis. It may even be possible to simultaneously obtain information on specific DNA and RNA targets via in situ hybridization. Similarly, several techniques have been recently developed to obtain simultaneous spatial and transcriptomic data, including multiplexed error-robust FISH (MERFISH) [146], laser capture microdissection sequencing (LCM-sequencing) [147], Tomo-seq [148], Slide-seq [149], and Spatial Transcriptomics [150]. These techniques may similarly be helpful in infectious disease to better define the interplay of immune cells, susceptible cells, stromal cells, and pathogens.

Another important gap that remains to be bridged is the ability to comprehensively access the state of a single cell across time. Both immune and infection processes are highly dynamic, but because most of the single-cell technologies listed above are destructive, changes over time must be assessed either by careful time-point studies, or by assuming the presence of a range of cells in a population that represent early and late stages of the process (e.g., cellular activation or infection stage). With the advent of microfluidic devices that can immobilize single cells for continued study, the same cell can be assessed at multiple points for longitudinal study. In addition to typical proteomic marker analyses and RNA or DNA in situ hybridization techniques with live cell imaging, it is already possible even to measure more complex phenotypes such as bioenergy metabolism [151]. Since microfluidic devices are also used for 3D organoid growth to simulate in vivo conditions, it is conceivable that future developments in technology will allow similar types of information to be collected in the context of organoids. This will represent one approximation toward high-dimensional in vivo data, which remains impossible with current methods.

Most of the single-cell studies reviewed in this article are based on end-point assays that are destructive and can therefore only measure a single time point of these single-cell targets. However, several recent studies outside the sphere of infectious disease have highlighted time as a prominent variable that influences the level of heterogeneity in host cells, immune cells, and pathogens. To that end, microfluidic platforms that enable the automated and precise control of media and reagents are ideal for performing such dynamic studies. For example, Wu et al. [152], using a customized microwell–microvalve system, performed a continuous measurement of A disintegrin and metalloproteinases (ADAMs) and matrix metalloproteinase (MMPs) secretions by single HepG2 cells upon a phorbol 12-myristate 13-acetate (PMA) challenge. Using their microfluidic platform, heterogenous changes in the secretion rates of ADAM and MMPs were observed in response to PMA stimulation, which may be used to predict HepG2 cell fates. In another recent study, a microfluidic platform that combined mutation visualization (MV) and microfluidic mutation accumulation (µMA) enabled real-time tracing of mutations of single bacteria [153].

Evidently, the utilization of microfluidic technology could enable high temporal resolution single-cell studies suited for uncovering the pathophysiology of infectious diseases. The advantages offered by microfluidics technologies include the efficient capture and compartmentalization of single cells, the precise control of fluid exchange, and ensuring a viable microenvironment for cell survival. These characteristics enable the dynamic study of large populations of single cells in parallel, which may eventually provide us with a more comprehensive understanding of the causes and effects of single-cell and single-pathogen heterogeneity.

## 7. Conclusions

Through the various applications of single-cell technology, we have gained a more thorough understanding of infectious disease pathophysiology at an unprecedented resolution. Revealing the heterogeneity within populations of pathogens has allowed a finer dissection of virulence factors, and similarly, heterogeneity within populations of infected cells has given us a deeper understanding of host immune defenses. In addition, the high-dimensional single-cell information that can be collected even from primary cells has allowed us to identify rare but important cell subtypes, and it has shed light on the complex interplay between the different cells of the immune system. With the advent of antibody and T cell-based therapeutics, and antibody-based diagnostics, the contributions of single-cell technology to the high-throughput identification of candidate B and T cell receptor sequences that are target-specific have also accelerated the development of new therapeutics and diagnostics for both newly emerging and existing diseases. The adoption of single-cell technologies is likely also to revolutionize clinical studies for both drugs and vaccines, given its immense potential for biomarker discovery. With the field of single-cell technology only just taking off in the last decade, there remain vast prospects in both the increased adoption of existing technologies and the development of new technologies.

## Figures and Tables

**Table 1:** Overview of commonly used single-cell technologies and their respective characteristics: a higher color intensity corresponds to a higher score (e.g., higher throughput, ease of moving cells of interest onto subsequent assays, higher information content, higher cost).

	Throughput	Downstream Assay Compatibility	Genetic Information	Epigenetic Information	Proteomic Information	Cell Function Information	Spatial Information	Temporal Information	Cost
*Bulk*									
Cell/organ function assays									
Next-Generation Sequencing (NGS)									
*Scalable to single cell*									
Polymerase Chain Reaction (PCR)									
Microfluidic tools									
Microscopy (including FISH)									
*Single cell*									
Flow cytometry (FACS)									
Mass Cytometry (CyTOF)									
Single Cell Sequencing									
CITE-seq/REAP-seq									
Imaging mass cytometry									
Spatial Transcriptomics									
Capability		**Not Able**		**Low**		**Medium**		**High.**	
